# Probing intracellular potassium dynamics in neurons with the genetically encoded sensor lc-LysM GEPII 1.0 in vitro and in vivo

**DOI:** 10.1038/s41598-024-62993-1

**Published:** 2024-06-14

**Authors:** Bernhard Groschup, Gian Marco Calandra, Constanze Raitmayr, Joshua Shrouder, Gemma Llovera, Asal Ghaffari Zaki, Sandra Burgstaller, Helmut Bischof, Emrah Eroglu, Arthur Liesz, Roland Malli, Severin Filser, Nikolaus Plesnila

**Affiliations:** 1 Institute for Stroke and Dementia Research (ISD), LMU University Hospital, LMU Munich, Munich, Germany; 2 Graduate School of Systemic Neurosciences, LMU Munich, Planegg-Martinsried, Germany; 3 Regenerative and Restorative Medicine Research Center (REMER), Research Institute for Health Sciences and Technologies (SABITA), Istanbul Medipol University, Istanbul, Turkey; 4 Molecular Biology, Genetics and Bioengineering Program, Faculty of Engineering and Natural Sciences, Sabanci University, Istanbul, Turkey; 5 Institut für Klinische Anatomie und Zellanalytik (Österbergstraße 3), Eberhard Karls Universität Tübingen, Tübingen, Germany; 6 Gottfried Schatz Research Center, Molecular Biology and Biochemistry, Medical University of Graz, Neue Stiftingtalstraße 6/4, 8010 Graz, Austria; 7 Department of Pharmacology, Toxicology and Clinical Pharmacy, Institute of Pharmacy, University of Tübingen, Auf der Morgenstelle 8, 72076 Tübingen, Germany; 8 Munich Cluster for Systems Neurology (SyNergy), Munich, Germany; 9 BioTechMed-Graz, Mozartgasse 12/II, 8010 Graz, Austria; 10 Deutsches Zentrum Für Neurodegenerative Erkrankungen (DZNE), Light Microscope Facility (LMF), Bonn, Germany

**Keywords:** Neuroscience, Fluorescence imaging

## Abstract

Neuronal activity is accompanied by a net outflow of potassium ions (K^+^) from the intra- to the extracellular space. While extracellular [K^+^] changes during neuronal activity are well characterized, intracellular dynamics have been less well investigated due to lack of respective probes. In the current study we characterized the FRET-based K^+^ biosensor lc-LysM GEPII 1.0 for its capacity to measure intracellular [K^+^] changes in primary cultured neurons and in mouse cortical neurons in vivo. We found that lc-LysM GEPII 1.0 can resolve neuronal [K^+^] decreases in vitro during seizure-like and intense optogenetically evoked activity. [K^+^] changes during single action potentials could not be recorded. We confirmed these findings in vivo by expressing lc-LysM GEPII 1.0 in mouse cortical neurons and performing 2-photon fluorescence lifetime imaging. We observed an increase in the fluorescence lifetime of lc-LysM GEPII 1.0 during periinfarct depolarizations, which indicates a decrease in intracellular neuronal [K^+^]. Our findings suggest that lc-LysM GEPII 1.0 can be used to measure large changes in [K^+^] in neurons in vitro and in vivo but requires optimization to resolve smaller changes as observed during single action potentials.

## Introduction

Potassium ions (K^+^) are the most abundant intracellular cations in most cell types and are involved in an array of vital processes both on the cellular and supracellular level^1^. These processes notably include the diffusion or transport of K^+^ across membranes to form an electrochemical gradient. Together with the electrochemical gradients of other ions, it makes up the membrane potential and can be utilized both as energy storage and for intracellular signaling^2^.

Dynamic shifts of intracellular K^+^ concentrations are fundamental during signal transduction by neurons, which transmit information via action potentials. After the initial depolarization phase of an action potential, the neuronal membrane is repolarized by the outflow of K^+^ through voltage-gated K^+^ channels or K^+^ leak channels to allow a new action potential to occur^3,4^. Therefore, the dynamics and the regulation of K^+^ ions play a crucial role in neurotransmission, and changes in brain K^+^ homeostasis are known to influence neuronal excitability^5,6^ as well as action potential shape^7,8^. In addition, restoration of the ion gradients after activity, mainly achieved via the Na^+^/K^+^-ATPase, is one of the primary energy-consuming processes in neurons, making ion homeostasis a key player in brain energetics and its regulation^9,10^. Perturbed K^+^ dynamics or mutations of K^+^ channels are directly responsible for many pathological conditions, such as epilepsy^11,12^ and migraine^13,14^. In contrast, other disorders, such as ischemic stroke, are often exacerbated or accompanied by perturbed K^+^ homeostasis^15^.

So far, technical limitations in measuring K^+^ fluxes in neurons have hampered our understanding of neuronal potassium dynamics. While electrode-based potassium measurements are fast and accurate, they are invasive, can measure K^+^ only very locally, and are primarily used to assess extracellular K^+^ dynamics^16,17^. Imaging methods such as MRI^18,19^ or live cell imaging with K^+^ sensitive dyes^20^ can potentially overcome these limitations; however, they lack (sub-)cellular resolution or specificity.

So far, technical limitations in measuring K+ fluxes in neurons have hampered our understanding of neuronal potassium dynamics. While electrode-based potassium measurements are fast and accurate, they are invasive, can measure K+ only very locally, and are primarily used to assess extracellular K+ dynamics16,17. Imaging methods such as MRI18,19 or live cell imaging with K+ sensitive dyes20 can potentially overcome these limitations; however, they lack (sub-)cellular resolution or specificity.

The development of genetically encoded fluorescent sensors for K^+^ ions holds great potential for overcoming those limitations, as they can be expressed in a cell-type-specific manner or with subcellular localization. In addition, they allow observation of K^+^ dynamics in single neurons and whole tissues non-invasively and can be measured with high temporal resolution. We and others^21^, recently developed novel FRET-based genetically encoded K^+^ ion indicators (GEPIIs) with high specificity. While these biosensors may help to understand K^+^ dynamics in the brain, they have been mainly tested in non-neuronal cells and have not been characterized neither in vitro nor in vivo for their feasibility to measure K^+^ dynamics in neurons.

Therefore, the aim of the current is to characterize the capacity of GEPIIs to observe K^+^ changes during neurotransmission, both in vitro and in vivo. To achieve this goal, we expressed the potassium sensor lc-LysM GEPII 1.0 in primary cultured neurons or in the cortex of living mice. We then recorded K^+^ signals of individual neurons during either spontaneous or evoked neuronal activity to assess the degree to which lc-LysM GEPII 1.0 can resolve K^+^ dynamics during neuronal activity.

## Methods

### Plasmids and cloning

We used plasmids carrying the genetically encoded potassium sensor lc-LysM GEPII 1.0 with different subcellular targeting sequences as previously described^21^. The sensor was targeted either to the cytosol (cyto), the plasma membrane (SubPM), or the mitochondria (mito). To achieve AAV-mediated expression, we subcloned the ORFs encoding the different variants of lc-LysM GEPII 1.0 in AAV backbones under the control of a CAG promoter for ubiquitous expression (pAAV-CAG-GFP, Addgene #37825), a hSyn promoter for neuronal expression (pAAV-hSyn-eGFP, Addgene #50465) or a GFAP promoter for astrocytic expression (pAAV.GFAP.iGABASnFr, Addgene #112172). All three backbones were linearized by cutting out the existing transgenes using BamHI and HindIII restriction sites. These restriction sites were added to cyto-lc-LysM GEPII 1.0 and SubPM-lc-LysM GEPII 1.0 via overhang PCR to insert them directly via subcloning. Since mito-lc-LysM GEPII 1.0 contains a BamHI restriction site, this transgene was inserted using the Gibson Assembly method. After cloning, DNA was transformed into competent DH5α *E. coli* bacteria, colonies were screened via colony PCR and candidate clones were confirmed by sequencing before amplification and AAV production.

pAAV.Syn.NES-jRCaMP1b.WPRE.SV40 was a gift from Douglas Kim & GENIE Project (Addgene plasmid # 100851; http://n2t.net/addgene:100851; RRID: Addgene_100851). For bacterial expression of jRCaMP1b, the construct was subcloned into EKAR2G_design1_mTFP_wt_Venus_wt vector (Addgene plasmid # 39813) using the restriction sites BamHI and XhoI.

For imaging the concentration of cytosolic calcium ions in vitro, we used pGP-AAV-syn-jGCaMP8m-WPRE, a gift from the GENIE Project (Addgene plasmid #162375)^22^. For optogenetic stimulation, we used pAAV-Syn-ChrimsonR-tdT, a gift from Edward Boyden (Addgene plasmid #59171)^23^.

### Protein purification

Bacterial expression plasmids containing lc-LysM GEPII 1.0 and jRCaMP1b were transformed into Rosetta (DE3) competent cells. For the selection of lc-LysM GEPII 1.0 and jRCaMP1b positive cells, kanamycin and ampicillin were used, respectively. For purification of the biosensors, positive clones were inoculated in 5 mL LB with appropriate antibiotic, and after 8 h, the cultures were transferred into 250 mL LB plus antibiotic and incubated at 37 °C. At an OD value between 0.4 and 0.6 protein expression was induced using IPTG with a final concentration of 0.5 mM and further incubated for 16 h at 18 °C. Purification of the 6 × histidine-tagged biosensors was performed using gravity-based Ni–NTA affinity chromatography method as described elsewhere^24^. Purified proteins were concentrated using Ultracel^®^ Regenerated Cellulose (30 kDa MWCO) Amicon tubes and kept at -80 °C for further usage. The functionality of the purified biosensors was tested using a SpectraMax i3 Multi-Mode Microplate Reader. Samples were loaded on a 96-well plate with a solid black bottom. The intensiometric calcium biosensors jRCaMP1b were excited at 535/25 nm and emission was collected at 595/35 nm. The FRET-based biosensors lc-LysM GEPII 1.0 were excited with 430/9 nm and emission was collected at 485/9–535/15 nm, respectively.

### Spectral analysis of purified proteins

Two-photon excitation and emission spectra of lc-LysM GEPII were recorded using a Leica SP8 DIVE 2-photon microscope equipped with a 25× water immersion objective. Purified proteins of lc-LysM GEPII 1.0 was diluted in a buffer containing 10 mM HEPES (pH7.4) and either 150 mM K^+^ or 150 mM Na^+^ and placed under the microscope. Excitation and emission spectra were recoded using the spectral analysis tool integrated in the Las X software (Leica).

Calibration curves of lc-LysM GEPII 1.0 were recorded by diluting purified protein in 10 mM HEPES buffer (pH7.4) with K^+^ concentrations varying from 150 to 0 mM. To avoid changes in buffer osmolality when reducing K^+^ concentrations below 150 mM, appropriate concentrations of Na^+^ were added to obtain a final concentration of both ions of 150 mM. These solutions were placed under the microscope, lc-LysM GEPII was excited at 850 nm, and fluorescence lifetime was recorded at 450–500 nm. Calibration curves of jRCaMP1b were recorded in a similar way. Solutions with different concentrations of Ca^2+^ were prepared using the Calcium Calibration Buffer Kit #1 (Cat: C3008MP, Thermo Fisher Scientific), jRCaMP1b was excited at 1045 nm, and fluorescence lifetime was recorded at 575–625 nm.

### AAV production

Adeno-associated viral particles were produced in HEK293T cells grown in DMEM supplemented with 10% FBS and 100 U/mL Penicillin–Streptomycin in a humidified incubator at 37 °C/5% CO_2_. Cells were grown to 70–80% confluency and triple transfected with pHelper, pAAV-DJ (both from Cell Biolabs, Cat: VPK-400-DJ) and a pAAV-ITR-vector carrying the transgene. Triple transfection was achieved using polyethylenimine (PEI) titrated to pH 7.0. Two to three days after transfection, cells were detached using 1/80 of the culture volume of 0.5 M EDTA in PBS pH 7.4 and AAV particles were extracted using the AAVpro^®^ Purification Kit (All serotypes) from Takara Bio Inc. (Cat: #6666). Cells were centrifuged at 1700*g* for 10 min at 4 °C and the supernatant was discarded. The resulting cell pellet was lysed by vortexing it with 650 µL of AAV Extraction Solution A plus. Subsequently, cell debris was pelleted at 14,000*g* for 10 min at 4 °C and the supernatant was collected in a new tube. Finally, 65 µL of AAV Extraction Solution B was added, and the viral solution was aliquoted and stored for further use at −80 °C. AAV titration was performed by qPCR using the AAVpro^®^ Titration Kit (for real-time PCR) Ver.2 from Takara (Cat: #6233) according to the manufacturer’s instructions. Titration was performed using primers that annealed in the ITR repeats of the viral backbone (ITR F: GGAACCCCTAGTGATGGAGTT and ITR R: CGGCCTCAGTGAGCGA).

### Preparation of cryo-stocks of mixed cortical cultures

Cryopreserved mixed cortical cell culture stocks were prepared from E17 embryos of Sprague Dawley rats. Brains were removed and immediately placed in ice-cold HBSS supplemented with 7 mM HEPES pH 7.4. Cortices were dissected, meninges were removed, and the tissue was cut into small pieces with a scalpel. Then the tissues from all embryos of one litter were digested in HBSS supplemented with 0.5% Trypsin and 10 µg/mL DNAse I for 15 min at 37 °C. Digestion was stopped by the addition of MEM supplemented with 10% FBS, the tissue was washed twice with HBSS and subsequently triturated using a glass Pasteur pipette coated with 4% BSA in HBSS. After counting the concentration of cells in the suspension, the cell suspension was diluted with MEM supplemented with 10% FBS to 2 million cells per mL. Finally, DMSO was added to a volume fraction of 10% and the solution was aliquoted. Aliquots were cryopreserved by placing them in a freezing container filled with isopropanol at −80 °C over night. The next morning, the aliquots were transferred into a liquid nitrogen tank for long-term storage.

### Mixed cortical cell cultures from cryo-stocks

All cell culture reagents were purchased from Gibco. Mixed cortical cultures were plated on 15 mm round glass cover slips coated with Poly-d-Lysine at a density of 100,000 cells per cover slip. A cryopreserved aliquot was thawed at 37 °C and diluted in an appropriate amount of culture medium (Neurobasal-A medium, no D-glucose, no sodium pyruvate supplemented with 1× B27, 10 mM glucose, 2 mM GlutaMAX™, 1 mM sodium pyruvate and 100 U/mL penicillin–streptomycin). The cell suspension was plated on the cover-slips placed in 12-well plates and maintained in a humidified incubator at 37 °C/5% CO_2_. Half of the culture medium was replaced twice a week and cells were used after 22–24 days. For transgene expression, cells were transduced 3–4 days before the experiment with the appropriate AAV at a MOI of 1000.

### Immunostainings

For immunocytochemistry, mixed cortical cultures were washed with PBS and subsequently incubated with 4% paraformaldehyde (PFA)/4% sucrose in PBS for 15 min at room temperature (RT). Fixed cells were washed three times with PBS, incubated with 50 µM NH_4_Cl in PBS for 10 min at RT, followed by another three washes with PBS. Fixed cells were then permeabilized using 0.1% TritonX in PBS for 3 min at RT and blocked with blocking buffer (0.2% BSA, 0.2% FCS, 0.02% fish-skin gelatin in PBS) for 1 h at RT. Subsequently, cultures were incubated with primary antibodies against gp-NeuN (Synaptic Systems, 266 004) and mouse-GFAP (Cell Signaling, #3670) diluted in blocking buffer for 2 h at RT. Subsequently, primary antibodies were removed, cells were washed three times with PBS for 5 min and then incubated with anti-guinea pig- AlexaFluor^®^ 647 (Jackson, 706-606-148) and anti-mouse AlexaFluor^®^ 594 (Jackson, 715-586-150) diluted in blocking buffer for 1 h at RT. During the last 5 min of this step, DAPI (5 µg/mL) was added to stain the nuclei. Finally, coverslips were washed three times with PBS for 5 min each and mounted on microscopy slides. Cells were imaged at a confocal microscope.

For immunohistochemistry, animals virally transduced with AAV-hSyn-lc-LysM GEPII 1.0 were anesthetized with MMF and transcardially perfused with PBS followed by 4% PFA in PBS until the liver was devoid of blood. Brains were extracted, post-fixed in 4% PFA in PBS overnight and then stored in PBS at 4 °C until further use. Brains were mounted in 4% agarose and sectioned using a vibratome to create 100 µm thick brain slices. PFA-fixed brain sections were incubated in a blocking and permeabilizing primary antibody buffer solution (1% BSA, 0.1% fish-skin gelatin, 0.1% Triton X-100, 0.05% Tween 20 in PBS) with rabbit anti-NeuN (Abcam, ab177487) antibodies at 1:100 dilution on a rotary shaker at 4 °C for 2–3 days. Sections were then washed in PBS three times for 30 min and incubated with a secondary antibody buffer mix (0.2% BSA, 0.2% FCS, 0.02% fish-skin gelatin in PBS) containing 1:300 anti-rabbit AlexaFluor^®^ 647 (Jackson, 711-606-152) antibody at 4 °C on a rotary shaker for two days. Sections were then washed three times in PBS and during the last washing step, DAPI was added at a concentration of 5 µg/mL for 30 min to stain the nuclei. Washing using PBS was repeated three times for 30 min prior to mounting the sections on glass coverslips. Brain section overviews were imaged with a confocal microscope using a 10× air objective.

### Live cell imaging

For live cell imaging of mixed cortical cultures expressing either GCaMP8m or lc-LysM GEPII 1.0, coverslips with the cultures were transferred in an open imaging chamber and placed on an inverted microscope. The microscope was equipped with a 20× air objective (NA 0.8, Ref: 420651-9911-000, Zeiss, Oberkochen, Germany), an LED light source (Colibri 7 R[G/Y]CBV-UV, Ref: 423052-9741-000, Zeiss, Oberkochen, Germany), an emission image splitter (Optosplit II, Cairn Research, Faversham, UK), and a CCD camera (Orca Flash 4.0, Ref: C11440-42U40 Hamamatsu Photonics, Hamamatsu, Japan). All optical filters and dichroic mirrors were purchased from Chroma (Bellows Falls, USA). Cells were constantly superfused with aCSF (in mM: 125 NaCl, 2.5 KCl, 1.25 NaH_2_PO_4_, 26 NaHCO_3_, 1 MgCl_2_, 1.25 CaCl_2_, 2 glucose, 0.5 sodium lactate, 0.05 sodium pyruvate) gassed with 5% CO_2_/95% air to maintain a stable pH at room temperature. GCaMP8m was excited with 10 Hz at 470 ± 20 nm and emission was collected with a bandpass filter at 525 ± 25 nm (ET470/40x; T495lpxr; ET525/50 m). For FRET imaging of lc-LysM GEPII 1.0, cells were excited at 436 ± 10 nm (ET436/20x; T455lp) and emitted light was split at 515 nm on the camera for simultaneous recording of mseCFP and cpV using a dichroic mirror (t515lp). Emission of the individual channels was collected using bandpass filters [480 ± 15 nm for mseCFP (ET 480/30 m), 535 ± 15 nm for cpV (ET 535/30 m)]. Depending on the experiment, cells expressing lc-LysM GEPII 1.0 were excited with frequencies ranging from 0.2 to 10 Hz and data was represented as FRET ratio cpV/mseCFP. Ratiometric imaging of SypHer3s was performed at 0.1 Hz using a double excitation, single emission paradigm. SypHer3s was excited at 405 ± 10 nm or 500 ± 10 nm and fluorescence was recorded at 535 ± 15 nm (ET405/20x; T425lpxr; ET535/30 m and ET 500/20x ; t515lp; ET 535/30 m, respectively). The pH signal was represented as the ratio of fluorescence recorded from excitation at 500 nm over that from excitation at 405 nm. Exposure time, LED power, and excitation frequencies were adjusted to minimize bleaching and phototoxicity while still obtaining sufficient signal-to-noise ratio.

### pH calibration

To obtain absolute pH values from experiments with SypHer3s, a pH calibration was performed in permeabilized neurons after each experiments. HEPES-buffered aCSF with high K^+^ concentrations (in mM: 127.5 KCl, 1.25 NaH_2_PO_4_, 10 HEPES, 1 MgCl_2_, 1.25 CaCl_2_) was used to mimic intracellular ionic levels. At the end of each experiment, high K^+^ ACSF equilibrated to different pH values ranging from 6.4 to 9.6 and supplemented with 10 µM Nigericin was superfused. The resulting SypHer3s ratios at each pH were fitted to a sigmoidal curve and the resulting function was used to calculate absolute pH values.

### K^+^ calibration

To calibrate lc-LysM GEPII 1.0, we permeabilized our cultures with 15 µM Valinomycin and applied 10 µM FCCP. Cultured neurons did not tolerate this protocol, therefore we performed the calibration in astrocytes. We applied aCSF (in mM: 1.25 NaH_2_PO_4_, 26 NaHCO_3_, 1 MgCl_2_, 1.25 CaCl_2_, 2 glucose, 0.5 sodium lactate, 0.05 sodium pyruvate) containing different concentrations of K^+^ ranging from 0 to 125 mM. To avoid changes in buffer osmolality when reducing K^+^ concentrations below 125 mM, appropriate concentrations of Na^+^ were added to obtain a final concentration of both ions of 125 mM.

### Optogenetic stimulation

For optogenetic stimulation, we co-transduced cultures with AAV-Syn-ChrimsonR-tdT and either AAV-CAG-lc-LysM GEPII 1.0, AAV-hSyn-SubPM-lc-LysM GEPII 1.0, or AAV-Syn-GCaMP8. Optogenetic stimulation was achieved by placing a red LED (M617L4, Thorlabs) over the cells during imaging using a custom-made 3D-printed holder. To maximize stimulation strength, the emitted light was collected using an aspheric condenser lens. A custom-made microcontroller-gated LED driver allowed programmable and precise light pulses for stimulation. Cells were stimulated with trains of 10 ms light pulses at 1 Hz, allowing enough time in between stimulation trains for complete recovery of the signal.

To avoid imaging artifacts from the stimulation light, a 590 nm long pass filter was placed in front of the LED and additional 550 nm short pass filters were added in the emission light path. The intensity of the excitation light for either GCaMP8m or lc-LysM GEPII 1.0 was minimized to avoid cross-stimulation of ChrimsonR. If the minimal LED power led to neuronal stimulation, it was further reduced using a neutral density filter OD 1.3.

### Animals

Two to three months old male C57BL/6J mice were used. The animals were group-housed under pathogen-free conditions and bred in the animal housing facility of the Center of Stroke and Dementia Research, with food and water provided ad libitum (21 ± 1 °C, at 12/12-h light/dark cycle). All experiments were carried out in compliance with the ARRIVE guidelines, the National Guidelines for Animal Protection, Germany and with the approval of the regional Animal Care Committee of the Government of Upper Bavaria.

### Cranial window implantation and stereotactic virus injection

Before use, surgical tools were sterilized in a glass-bead sterilizer (Fine Science Tools). Mice were anesthetized by an i.p. injection of medetomidine (0.5 mg/kg), midazolam (5 mg/kg), and fentanyl (0.05 mg/kg). Subsequently, mice were placed onto a heating blanket (37 °C), and the head was fixed in a stereotactic frame. Eyes were protected from drying by applying eye ointment. The scalp was washed with swabs soaked with 70% ethanol. A flap of skin covering the cranium was excised using small scissors. The periosteum was scraped away with a scalpel. The prospective craniotomy location over the somato-sensory cortex was marked with a biopsy punch (diameter 4 mm). The exposed skull around the area of interest was covered with a thin layer of dental acrylic (iBond Self Etch, Heraeus Kulzer) and hardened with an LED polymerization lamp (Demi Plus, Kerr). A dental drill (Schick Technikmaster C1, Pluradent) was used to thin the skull around the marked area. After applying a drop of sterile phosphate buffered saline on the craniotomy, the detached circular bone flap was removed with forceps. Subsequently, 0.5 µl of a virus suspension (AAV.PhPeb-Syn.NES- lc-LysM GEPII 1.0, Vectorbuilder and AAV.9-Syn.NES-jRCAMP1b, Addgene #100851-AAV9 mixed 1:1 to a final concentration of 1 × 10^12^ vg/ml) was injected via a glass capillary and Nanoliter 2020 Injector (World Precision Instruments) at a speed of 50 nl/min into the somatosensory cortex at the following coordinates: −1.5 mm rostrocaudal, −1.5 mm lateral and −0.25 mm dorsoventral. A circular coverslip (4 mm diameter) was placed onto the craniotomy and glued to the skull with histoacryl adhesive (Aesculap). The exposed skull was covered with dental acrylic (Tetric Evoflow A1 Fill, Ivoclar Vivadent), and a head-post was attached parallel to the window for head-fixing mice in subsequent imaging sessions. After surgery, mice received a s.c. dose of the analgesic Carprophen (7.5 mg/kg body weight). Anesthesia was antagonized using Atipamezol (2.5 mg/kg), Naloxone (1.2 mg/kg), and Flumazenil (0.5 mg/kg) i.p. Finally, mice were allowed to recover in a 35 °C warming chamber until full recovery. In vivo imaging was commenced 3 to 4 weeks after surgery.

### Mouse cerebral ischemia model

For remote occlusion of the middle cerebral artery (MCAo) in mice^25^ the left common carotid artery was exposed and the superior thyroid artery branching was cauterized. The common carotid artery was ligated and the external carotid artery (ECA) was fully occluded. A filament with a diameter of 0.21 ± 0.02 mm (6021PK10, Doccol Corporation, Sharon, MA, USA) was inserted through as small incision into the ECA and advanced for 5 mm. A custom-made occlusion filament was inserted into the ECA while the Doccol filament was simultaneously removed. The filament was then further advanced along the internal carotid artery towards the middle cerebral artery (MCA). After placing the under the 2-photon microscope the MCA could be occluded remotely. MCA occlusion (MCAo) caused a cortical infarct which induced periinfarct depolarizations waves (PIDs) within the surrounding tissue.

### In vivo two-photon microscopy

In vivo two-photon imaging was performed three weeks after cranial window implantation using a Leica SP8 DIVE 2-photon microscope equipped with a fs-laser, a 25× water immersion objective and a motorized stage. lc-LysM GEPII 1.0 and jRCaMP1b were co-excited at 850 nm and 1045 nm, respectively, and the emission was collected at 450–500 nm and 575–625 nm with non-descanned detectors placed directly behind the objective. Laser power below the objective was kept around 50 mW to minimize phototoxicity. Throughout the imaging session, mice were anesthetized with isoflurane (1% in oxygen, 0.5 l/min) and kept on a heating pad to maintain body temperature at 37 °C. XY time-lapse series (7.51 Hz) with 1 µm axial resolution and 128 × 128 pixels per image frame of lc-LysM GEPII 1.0 and jRCaMP1b expressing neurons were subsequently recorded at a depth of 150–200 µm underneath the cortical surface. After 5 min of baseline recordings, periinfarct depolarizations were induced via remote MCAo and recordings were continued for further 10 min. Fluorescence lifetime measurements were subsequently analyzed in LAS X (Leica).

### Data processing

Bleaching of experiments using lc-LysM GEPII 1.0 was corrected using a custom-written Python script by fitting data of the individual channels to exponential decay curves. Only data points that were visually identified as baseline (before stimulus application and after full signal recovery) were used for the fit. The resulting parameters were used to calculate corrected donor and acceptor channels, from which the corrected FRET ratio was calculated.

Calculations of the standard deviation for noise quantification was performed from 1000 frames of experiments recorded at 10 Hz. Since some traces of GCaMP8m displayed slow baseline shifts interfering with SD calculation, smaller blocks of the traces were individually normalized every 17 frames to correct for these shifts. This approach did not mask any signals evoked by neuronal activity. SD was calculated from the corrected trace. For consistency, the same approach was used for traces recorded with lc-LysM-GEPII 1.0, which did not change the results compared to analysis of the full trace standard deviation. SD values were plotted against the intensity of the corresponding cell. A power function was fitted to the data to quantify the correlation strength.

Amplitudes of the lc-LysM GEPII 1.0 response to optogenetic stimulations were calculated as the FRET ratio at baseline (FRET_BL_) minus the mean FRET ratio of the last two frames of the respective stimulation (FRET_stim_). Baseline was calculated as the mean of the FRET ratio of the 20 frames before stimulation onset. All neurons per experiment were averaged to minimize bias by batch effects from experiments with a high number of neurons, and the mean of all experiments was used to determine the presence or absence of a response. Two-tailed, paired t-tests were performed between FRET_BL_ and FRET_stim_ to determine if a specific stimulation elicited a significant change (p < 0.05) in the FRET signal of lc-LysM GEPII 1.0.

## Results

After confirming that we were able to successfully express lc-LysM GEPII 1.0 in a cell type-specific manner and to target different subcellular compartments (see supplementary Fig. 1), we explored the potential for resolving K^+^ changes during neuronal activity by live cell imaging. Ca^2+^ dynamics were measured in parallel experiments using GCaMP8m to monitor neuronal activity. lc-LysM GEPII 1.0 fluorescence was measured in rat primary mixed cortical cultures containing neurons and astrocytes.

lc-LysM GEPII 1.0 resolves [K^+^] decreases in response to tetanic neuronal stimulation in vitro.

**Figure 1:**
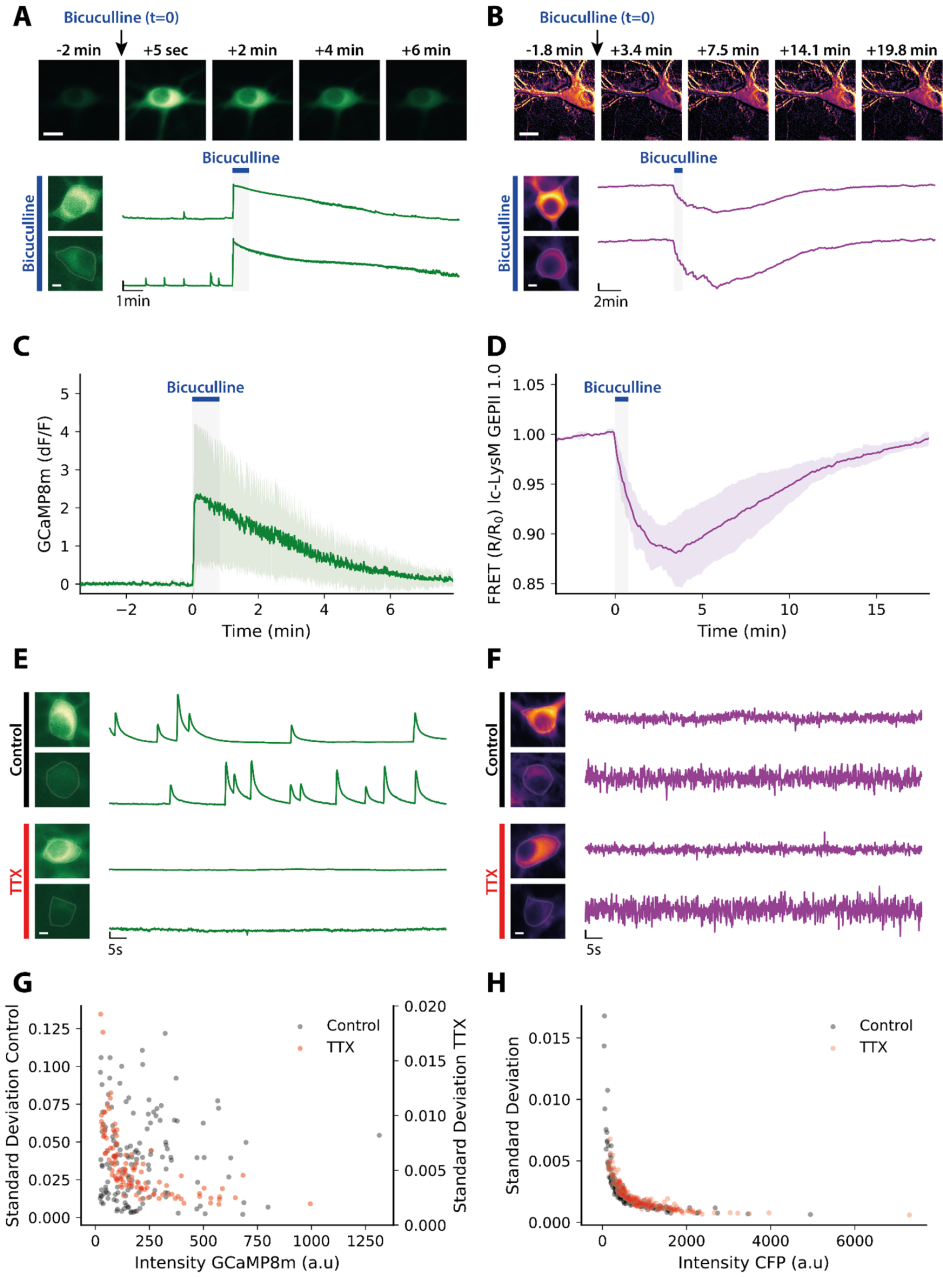
lc-LysM GEPII 1.0 resolves neuronal potassium changes only upon strong stimulation. **(A)** Neurons expressing GCaMP8m were treated with 50 µM Bicuculline for 50 s. The time series shows a lasting increase of the intensity of GCaMP8m. Scale bar represents 15 µm. Below are example traces of the response to Bicuculline of two individual neurons with different intensities of GCaMP8m. Horizontal scale bar: 5 µm, vertical scale bar 0.5 [dF/F]. **(B)** Bicuculline leads to a decrease of the FRET ratio of lc-LysM GEPII 1.0. Images are pseudocolored according to the FRET ratio. Horizontal scale bar: 5 µm, vertical scale bar: 0.05 [normalized FRET ratio]. **(C)** Average trace of the GCaMP8m response to Bicuculline (N = 4 experiments). Traces are represented as mean ± SD. **(D)** Averaged trace of the lc-LysM GEPII 1.0 response to Bicuculline(N = 5 experiments). Traces represented as mean ± SD. **(E)** Calcium traces of individual neurons with either high or low baseline intensity [horizontal scale bar: 5 µm, vertical scalebar: 0.2 (dF/F)]. Neurons under control conditions show clear spontaneous activity, which is completely inhibited in the presence of 500 nM TTX. **(F)** Potassium traces of individual neurons with either high or low baseline intensity [horizontal scale bar: 5 µm, vertical scale bar: 0.005 (normalized FRET ratio)]. No obvious signal above noise is observed and no difference between control condition and 500 nM TTX can be detected. Noise is considerably bigger in cells with lower baseline intensity of lc-LysM GEPII 1.0. **(G)** Standard deviation of the traces recorded from neurons expressing GCaMP8m plotted against baseline intensity under various conditions. No clear correlation can be observed for the control condition (grey, 142 neurons in 8 experiments) or Bicuculline treatment (blue, 104 neurons in 4 experiments). In contrast, neurons silenced with TTX show a clear correlation between SD and intensity (red, 112 neurons in 7 experiments). **(H)** Standard deviation of the traces recorded from neurons expressing lc-LysM GEPII 1.0 plotted against the baseline intensity of the donor. There is no difference between neurons under control conditions (grey, 152 neurons in 13 experiments) and in the presence of TTX (red, 192 neurons in 13 experiments). Both cases show a clear correlation of standard deviation with intensity. For Bicuculline, standard deviation does not correlate with the intensity of CFP (blue, 45 neurons in 5 experiments).

During neuronal activity, neurons depolarize due to the inflow of sodium (Na^+^) and calcium ions (Ca^2+^) and repolarize due to the outflow of K^+^. Our goal was to investigate if and to what extent we could observe this K^+^ outflow during neuronal activity using lc-LysM GEPII 1.0. Therefore, as a proof-of-principle experiment, we induced neuronal hyperactivity in our mixed cortical cultures by inhibiting inhibitory neurons with Bicuculline, a selective GABA_A_ receptor antagonist. Ca^2+^ imaging confirmed that Bicuculline induced a strong, epileptic-like neuronal firing pattern. The addition of Bicuculline for 50 s led to an immediate increase of the intensiometric signal of GCaMP8m, which peaked within seconds (Fig. 1A, C). After Bicuculline was washed out, the Ca^2+^ signal recovered to baseline. When we applied the same stimulus (50 µM Bicuculline for 50 s) to cultures expressing lc-LysM GEPII 1.0, we observed a pronounced decrease of the FRET ratio signal by 12.7 ± 3.4%, indicating a reduction in the intracellular K^+^ concentration (Fig. 1B, D). Neuronal activity induced by Bicuculline led to a pronounced cytosolic acidification. The pH response reached its minimum after 2.2 ± 0.4 min before recovering to baseline (supplementary Fig. 2A, B). In contrast, the FRET ratio signal of the K^+^ biosensor continued to decrease during initial wash-out and reached a minimum after 3.5 ± 0.5 min, while pH already started to recover. In addition, the individual donor and acceptor signals of lc-LysM-GEPII 1.0 show opposite behavior during Bicuculline application, implying a real FRET effect rather than interference by pH (supplementary Fig. 2C, D). We obtained similar results when inducing epileptiform activity using the non-selective inhibitor of voltage-dependent K^+^-channels 4-Aminopyridine, which led to a delayed decrease in the FRET ratio of the lc-LysM GEPII 1.0 (supplementary Fig. 3). Together, these data show that induced epileptiform activity is associated with reduced cytosolic K^+^ levels and demonstrates the suitability of the FRET-based biosensor to monitor K^+^ changes in response to neuronal hyperactivity.

### Measuring lc-LysM GEPII 1.0 fluorescence during spontaneous neuronal activity in cultured neurons

After confirming that we can resolve cytosolic [K^+^] alterations during intensive neuronal activity associated with large changes in intracellular ion concentrations, we aimed to investigate if we could observe K^+^ changes during spontaneous neuronal activity characterized by mainly single action potentials. Ca^2+^ imaging using GCaMP8m showed that our cultures displayed spontaneous neuronal activity, which was completely abolished in the presence of tetrodotoxin (TTX), a potent inhibitor of voltage-gated sodium channels (Fig. 1E). When measuring lc-LysM GEPII 1.0 fluorescence, the obtained recordings did not display any apparent changes of the FRET ratio signal (Fig. 1F). When inhibiting neuronal activity using TTX, the FRET ratio traces were indistinguishable from the ones before TTX application (Fig. 1F). To assess whether the lc-LysM GEPII 1.0 FRET ratio signals were due to neuronal activity or technical noise, we hypothesized that changes in the FRET ratio signal in response to actual K^+^ fluctuations should be independent of signal intensity. In contrast, since the relative contribution of technical noise to the measured signal is bigger for less bright signals, FRET changes due to noise should correlate with signal intensity. To test this hypothesis, we plotted the standard deviation (SD) of the full calcium traces against the average intensity of GCaMP8m. Indeed, our analysis showed no correlation between SD and signal intensity in spontaneously firing neurons (Fig. 1G, gray symbols, R^2^ = 0.005) indicating that neuronal activity induced changes in the Ca^2+^ signal disturb the correlation between SD and intensity. In contrast, for neurons silenced with TTX these parameters correlated well (Fig. 1G, orange symbols, R^2^ = 0.71) suggesting that the recorded signal represented background noise. Performing the same analysis for signals derived under the same conditions from neurons expressing lc-LysM GEPII 1.0, we observed no changes between the control condition, i.e., when neurons fired spontaneously (Fig. 1H, gray symbols, R^2^ = 0.92) and the time when spontaneous neuronal activity was inhibited by TTX (Fig. 1H, orange symbols, R^2^ = 0.91). These data indicate that neuronal activity did not contribute to the FRET ratio signal changes of lc-LysM GEPII 1.0., i.e., the recorded signal was background noise. A calibration of lc-LysM GEPII 1.0 in permeabilized astrocytes yielded an EC_50_ for K^+^ of 35 mM and showed that lc-LysM GEPII 1.0 is saturated or close to saturation at baseline K^+^ levels (supplementary Fig. 4). These findings suggest that lc-LysM GEPII 1.0 is not able to measure changes in intracellular [K^+^] elicited by single action potentials or, alternatively, that single action potentials may not result in global reductions of cytosolic [K^+^].

### lc-LysM GEPII 1.0 signals following optogenetic stimulation of neurons

**Figure 2:**
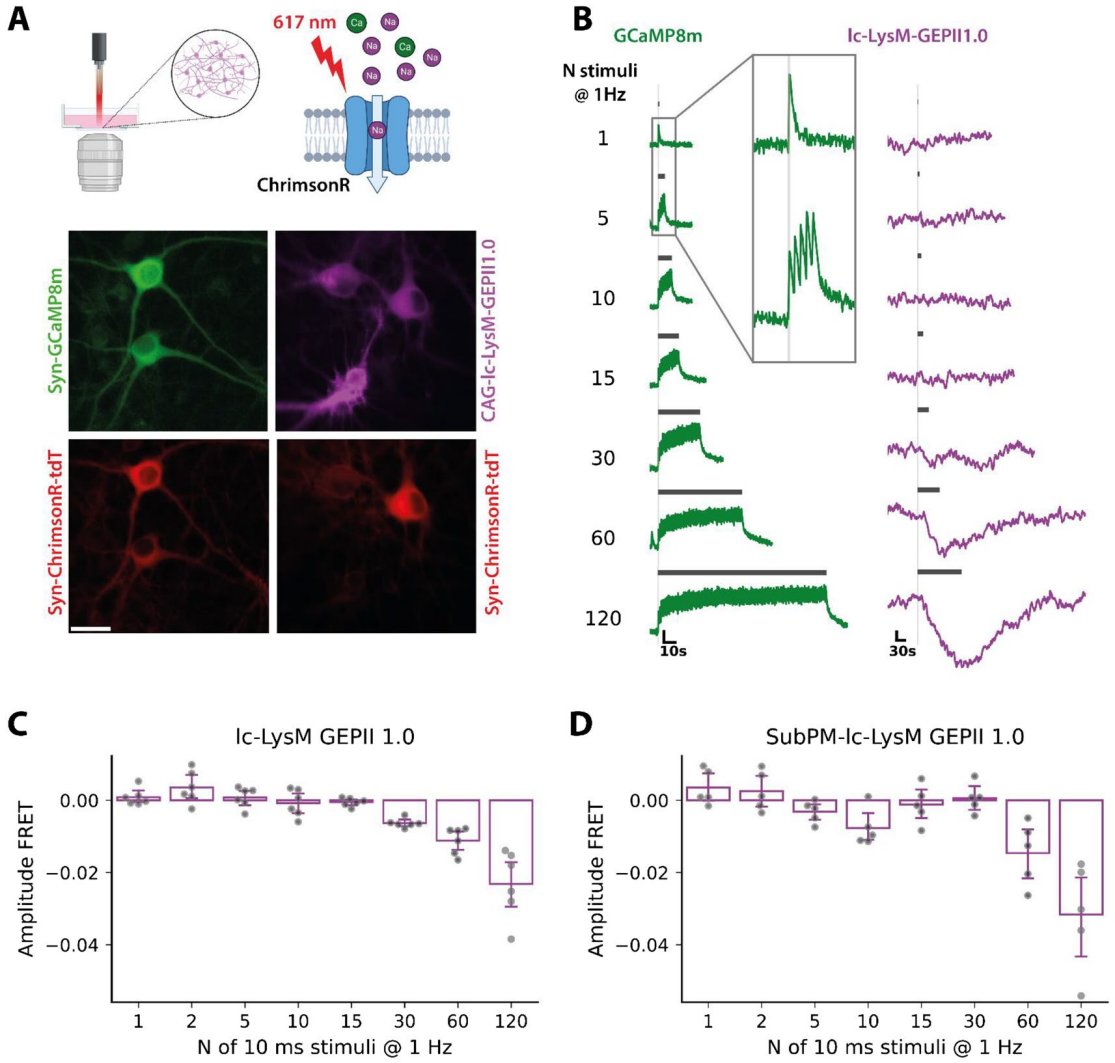
Optogenetic stimulation to fine-tune responsivity of lc-LysM GEPII 1.0. **(A)** Schematic illustration of the setup for optogenetic stimulation of ChrimsonR during live cell imaging (created with BioRender.com). Cells are illuminated with a LED with an emission peak at 617 nm. Cells were co-transduced with AAV-Syn-GCaMP8m and AAV-Syn-ChrimsonR-tdT (left) or AAV-CAG-lc-LysM GEPII 1.0 and AAV-Syn-Chrimson-tdT (right) to allow imaging of calcium in neurons or potassium in neurons and astrocytes during neuronal stimulation respectively. Scale bar represents 25 µm. **(B)** Cells were stimulated with 10 ms pulses at a stimulation rate of 1 Hz. Representative traces of GCaMP8m [left, vertical scale: 0.2 (dF/F)] and lc-LysM GEPII 1.0 [right, vertical scale: 0.05 (normalized FRET ratio)] in response to different amounts of pulses. Each pulse elicited a calcium spike indicating successful and robust stimulation. Potassium changes were only visible after a train of 30 or more stimulations. **(C)** Quantification of the amplitudes of the normalized FRET ratio of lc-LysM GEPII 1.0 expressed in neurons in response to an increasing number of stimuli. A drop of neuronal potassium levels was observed starting at 30 stimulations and increased with the number of additional stimulations. Amplitudes were quantified as the difference of the FRET ratios between the baseline before the stimulation and the mean of the last two frames of each train of stimulation. Data is represented as mean ± SD (N = 46 neurons in n = 6 experiments). **(D)** Quantification of the amplitudes of the normalized FRET ratio of lc-LysM-GEPII1.0 targeted to the plasma lemmal space of neurons. Data is represented as mean ± SD (N = 25 neurons in n = 5 experiments).

Having identified that lc-LysM GEPII 1.0 can resolve K^+^ decreases in response to multiple but not to single action potentials, we wanted to investigate the threshold above which lc-LysM GEPII 1.0 is able to resolve detectable [K^+^] changes in neurons. We combined optogenetic neuronal stimulation with FRET ratio imaging of lc-LysM GEPII 1.0, allowing us to control neuronal activity during live imaging of [K^+^] changes. We chose the red-shifted optogenetic tool ChrimsonR to avoid cross-stimulation with the excitation light during imaging and co-expressed it with either GCaMP8m or lc-LysM GEPII 1.0 (Fig. 2A). We decided to stimulate our mixed cortical cultures using low-frequency stimulation trains with increasing pulses to investigate the minimal activity required for detecting K^+^ changes using lc-LysM GEPII 1.0. We chose 10 ms as the individual pulse length as this was the shortest duration reliably eliciting a Ca^2+^ signal during imaging with GCaMP8m (see supplementary Fig. 5). We stimulated neurons with up to 120 light pulses and allowed the signal to return to baseline before triggering the next stimulation train. Each stimulation triggered an individual peak of [Ca^2+^]_i_ as evidenced by imaging with GCaMP8m (Fig. 2B, green traces); when measuring potassium using lc-LysM GEPII 1.0 we could observe a decrease of FRET ratio signals (normalized FRET change: −0.63 ± 0.12%, p < 0.001) only after a stimulation train of 30 pulses (Fig. 2B, purple traces and Fig. 2C), again confirming that multiple action potentials are necessary to decrease global cytosolic [K^+^] to levels detectable with lc-LysM GEPII 1.0. Increasing the number of pulses per stimulation train to 60 or 120 further reduced FRET ratio signals of the K^+^ biosensor (normalized FRET changes −1.12 ± 0.37%, p < 0.001 and −2.31 ± 0.93%, p < 0.001, respectively) linearly (R^2^ = 0.997). Interestingly, when we targeted lc-LysM GEPII 1.0 to the plasmalemmal space of neurons and performed the same stimulation paradigm (Fig. 2D), we only observed decreases in the FRET ratio after 60 or 120 stimuli (−1.46 ± 0.86%, p = 0.02 and 3.16 ± 1.47%, p = 0.008, respectively), however, not after 30 stimuli (p = 0.77). In addition, we observed a decrease after 10 stimuli (−0.0077 ± 0.0051, p = 0.028).

### lc-LysM GEPII 1.0 allows measuring [K^+^]_i_ in neurons in vivo

**Figure 3:**
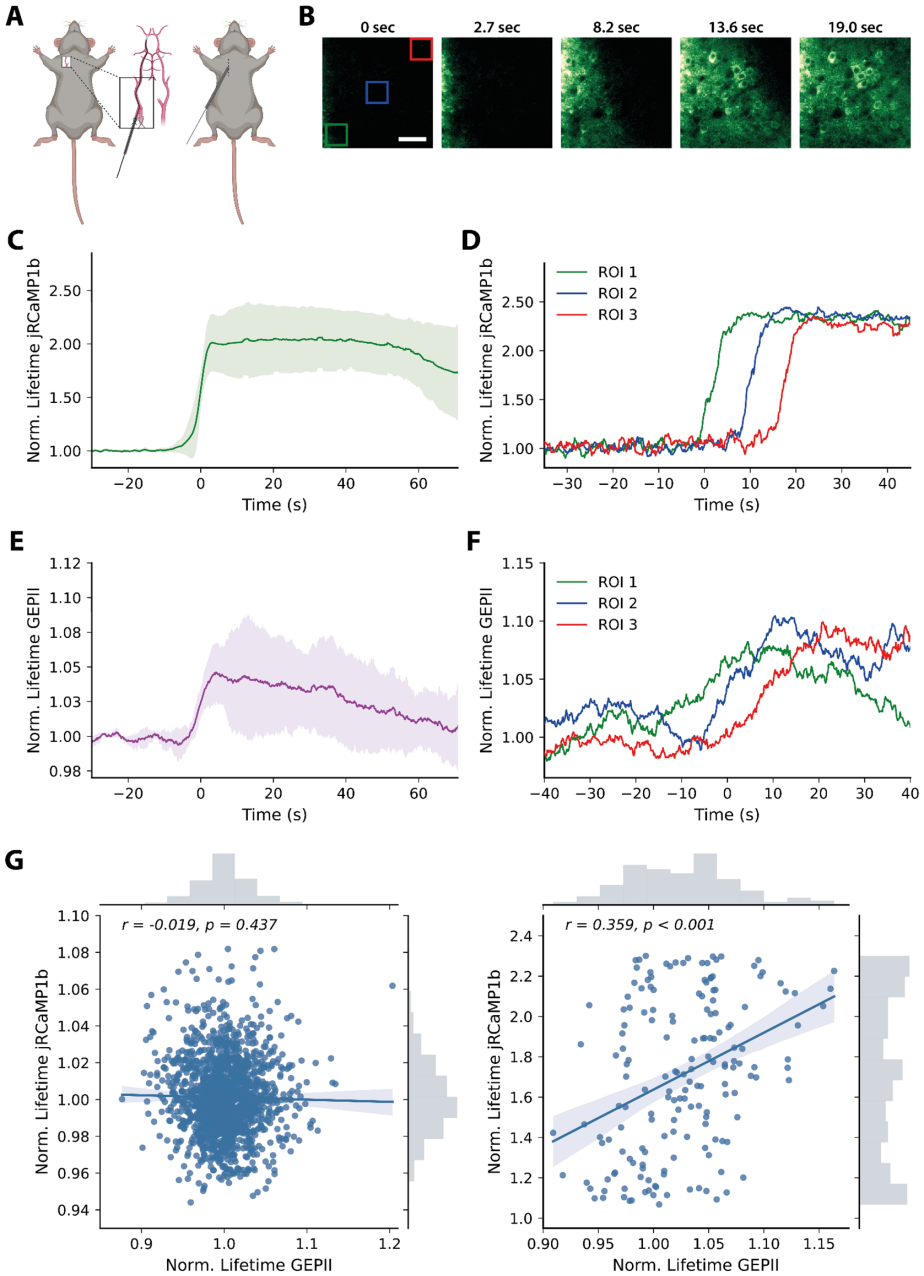
In vivo imaging of lc-LysM GEPII 1.0 during a peri-infarct depolarization in the cortex of living mice. **(A)** Schematic illustration of the surgical procedure (created with BioRender.com). Peri-infarct depolarizations (PID) were elicited via insertion of a filament into the external carotid artery and subsequent occlusion of the middle cerebral artery during the imaging session. The middle cerebral artery was kept occluded throughout the experiment. **(B)** Pseudocolored image strip shows the intensity of jRCaMP1b during a PID over time. Neuronal calcium levels increase in a synchronized wave like manner. **(C)** Average FLIM response of jRCaMP1b during a PID show a robust increase of the fluorescence lifetime. Data is represented as mean ± SD. To overlap PIDs from individual experiments (N = 3), we set the half-maximal FLIM increase t = 0. **(D)** FLIM responses of individual regions of interest (indicated in **3B**) placed along the field of view of a single recording show the wave-like character of the PID. **(E)** Average FLIM responses of lc-LysM GEPII 1.0 during PIDs (N = 3) show an increase of the fluorescence lifetime of lc-LysM GEPII 1.0, indicating a decrease of the neuronal potassium levels. Data represented as mean ± SD. **(F)** FLIM of lc-LysM GEPII 1.0 also shows the wave-like character of the PID. **(G)** Correlation of the normalized lifetimes of jRCaMP1b and lc-LysM GEPII 1.0 during baseline imaging (left) and during the rising phase of the CSD (right). No significant correlation during baseline imaging could be observed whereas during the CSD, increases of the lifetime of jRCaMP1b correlate significantly with increases of lc-LysM GEPII 1.0

After having characterized lc-LysM GEPII 1.0 in cultured cells, we wanted to investigate if we can also measure neuronal K^+^ dynamics in the brain of living mice by expressing hSyn-lc-LysM GEPII 1.0 and Syn-jRCaMP1b with AAV-based viral vectors in the cerebral cortex (supplementary Fig. 6). Since we observed changes of cytosolic [K^+^] only in response to intense neuronal activity in vitro, we expected a similar situation in vivo. To generate vigorous neuronal activity in vivo, we chose a model resulting in massive depolarizations of all neurons within a given spatial and temporal window. For this purpose, we induced focal cerebral ischemia by remote occlusion of the middle cerebral artery (MCAo; Fig. 3A), a condition well known to cause waves of massive neuronal depolarizations in the vicinity of ischemic tissue, so called periinfarct depolarizations (PIDs) while mice were placed under a 2-photon microscope for simultaneous recordings of jRCaMP1b and lc-LysM GEPII 1.0. Within minutes of MCA occlusion, PIDs were recorded in the mouse cerebral cortex as evidenced by increases in neuronal [Ca^2+^]_i_ spreading in cortical tissue with the expected velocity of about 2 mm/min (Fig. 3B). As a PID also elicits an increase in cerebral blood flow that may interfere with intensity-based fluorescent imaging approaches, we decided to measure jRCaMP1b and lc-LysM GEPII 1.0 using fluorescence lifetime imaging (FLIM), which is independent of intensity changes and therefore provides a robust readout. PIDs elicited a substantial increase in the fluorescence lifetime of jRCaMP1b by 111 ± 30% (Fig. 3C), crossing the field of view (Fig. 3D). Having now established in vivo FLIM microscopy, we used the same experimental protocol to check if we could measure [K^+^] changes during PIDs using lc-LysM GEPII 1.0. For this purpose, we measured the fluorescence lifetime of the donor mseCFP as a proxy of the FRET efficiency of the sensor. After induction of PIDs by cerebral ischemia, we recorded an increase in the fluorescence lifetime of the donor fluorescence of lc-LysM GEPII 1.0 by 5.9 ± 2.4% (Fig. 3E). When placing regions of interest in different parts of the field of view, we observed the wave-like character of the PID, confirming that the signal increase occurred in response to the PID (Fig. 3F). An increased fluorescence lifetime usually corresponds to a decrease of the FRET efficiency of a sensor as energy transfer from the excited donor fluorophore is less efficient and therefore slower. Indeed, when we calibrated the fluorescence lifetime changes of lc-LysM-GEPII 1.0 in response to different K^+^ concentrations using purified protein, we observed that an increase in the lifetime corresponds to a decrease of neuronal [K^+^] (supplementary Fig. 7), as is expected during strong neuronal depolarization. As lifetime changes are not affected by intensity or potential volume changes during the PID, confounders are unlikely to account for this effect. When we correlated the normalized fluorescence lifetime values of lc-LysM GEPII 1.0 with those of jRCaMP1b, we could not observe any correlation between the two sensors before PID induction (R = −0.019, p = 0.437, Fig. 3G). This confirms that we cannot resolve intracellular [K^+^] changes using lc-LysM GEPII 1.0 during spontaneous neuronal activity. However, an analogous correlation between the FLIM values for lc-LysM GEPII 1.0 and jRCaMP1b during the rising phase of the PID revealed a highly significant correlation (R = 0.359, p < 0.001, Fig. 3G). The range of FLIM values of jRCaMP1b is considerably more extensive during PIDs, indicating that a very intense neuronal activation is required to elicit changes in [K^+^]_i_ big enough to be detected by lc-LysM GEPII 1.0. These data strongly suggest that we managed for the first time to record neuronal [K^+^] changes in the brain of living mice.

## Discussion

In the current study, we characterized the functionality of the FRET-based genetically encoded K^+^ sensor lc-LysM GEPII 1.0 in neuronal cells with a specific focus on measuring changes of [K^+^]_i_ during neuronal activation. We provide proof-of-principle evidence that lc-LysM GEPII 1.0 can resolve differences in [K^+^]_i_ during intense neuronal activity in cultured cells and in vivo. Changes of [K^+^]_i_ during single action potentials, as observed during spontaneous neuronal activity, could not be detected either in vitro or in vivo. However, we could successfully measure [K^+^]_i_ changes in the mammalian brain in vivo using the FRET-based K^+^ biosensor if massive neuronal activity was induced.

Brain potassium homeostasis is crucial for proper neuronal function, including controlling neuronal excitability or neurovascular and -metabolic coupling^26^ and its disruption, e.g., through mutations in K^+^ channels are involved in severe neurological pathologies such as epilepsy^12^ or migraine^27^. Therefore, precise monitoring of K^+^ dynamics in brain tissue would be desirable to understand better K^+^ fluxes and how they are controlled. However, this understanding has been hampered by technical challenges.

The emergence of genetically encoded sensors for potassium^21,28–30^ sparked hope that imaging approaches can help answer questions that could not be tackled with other methods^5,31,32^. However, applications of genetically encoded K^+^ sensors to record intracellular neuronal potassium dynamics are sparse. One possible reason is that available sensors have a very high affinity for K^+^ and are saturated when expressed in neurons, cells which have a very high intracellular K^+^ concentration of around 140 mM^9^. For the current study, we chose lc-LysM GEPII 1.0, which, at the start of the study, had the highest K_d_ of all available sensors (i.e., 27 mM in vitro and 60 mM in cells^21,28^). In the meantime, a new generation of K^+^ biosensors optimized for intracellular recordings has been published, yet they still need to be investigated in neurons^30^.

When we induced hyperactivity in our neuronal cultures using Bicuculline or intensely stimulated these cells optogenetically (at least 30 times at 1 Hz), we observed a decrease of the FRET ratio of lc-LysM GEPII 1.0, indicating a reduction in neuronal [K^+^]_i_. This is expected, as K^+^ outflow is the main contributor to neuronal repolarization after an action potential and was also corroborated by all so far published studies measuring intracellular K^+^ dynamics in neurons using genetically encoded sensors independent of the stimulation paradigm^28,29,33^. Before concluding that lc-LysM GEPII 1.0 indeed measures [K^+^]_i_ one has to consider that next to changes in [K^+^]_i_ strong neuronal activity also leads to an intracellular acidification by up to 0.3 pH units^34,35^. Since genetically encoded sensors are based on fluorescent proteins prone to pH-induced changes, intracellular pH shifts may result in apparent changes in [K^+^]_i_. Wu and colleagues suggested that the decrease of the signal of the K^+^ sensor GINKO2 was, to a large degree, caused by acidification of the neuronal cytoplasm rather than K^+^ changes^29^. The specific pH stability of the K^+^ indicator used in the current study, i.e., lc-LysM GEPII 1.0, has not been evaluated; however, lc-LysM GEPII 1.0 is derived by only 3 point mutations from GEPII 1.0, which shows only negligible pH sensitivity in the physiological range^21^. Furthermore, the fluorescence of the individual donor and acceptor channels changed in opposite directions thereby strongly suggesting a real signal. Therefore, it is reasonable to conclude that the decrease of the FRET ratio of lc-LysM GEPII 1.0 in response to Bicuculline observed in the current study is indeed caused by a reduction in [K^+^]_i_. Hence, we can conclude with a reasonable confidence level that lc-LysM GEPII 1.0 accurately detects [K^+^]_i_ during neuronal activity.

After ensuring the general functionality of lc-LysM GEPII 1.0 in neurons, we aimed to investigate whether the biosensor can detect changes of [K^+^]_i_ occurring during single action potentials. Our cultured cortical neurons displayed spontaneous neuronal activity consisting of multiple single action potentials as evidenced by single, sharp increases in [Ca^2+^]_i,_ which could be silenced with the inhibitor of voltage-gated sodium channels TTX. After characterizing these robust spontaneous [Ca^2+^]_i_ fluctuations in our cell culture system, we transduced our cells with lc-LysM GEPII 1.0. We could not observe any distinguishable changes in the lc-LysM GEPII 1.0 signals that indicated spontaneous K^+^ dynamics. In addition, the signal was not affected by TTX-induced neuronal silencing and correlated with the expression level of the sensor. These findings suggest that lc-LysM GEPII 1.0 failed to detect [K^+^]_i_ changes induced by single action potentials. This conclusion is further supported by the observation that controlled optogenetic stimulation of neurons with low frequencies also failed to elicit any change in lc-LysM GEPII 1.0 fluorescence.

These data may be interpreted in three ways: 1) either lc-LysM GEPII 1.0 is not sensitive enough to resolve small changes of [K^+^]_i_, or 2) up to a certain number of action potentials [K^+^] does not decrease in the cytoplasm or decreases only in the vicinity of the cell membrane, or 3) both scenarios are valid. To understand which of these scenarios is the most likely, it is essential to know that [K^+^]_i_ in neurons is around 140 mM while in the extracellular space, it is only 3 mM^6^. In addition, the extracellular space only makes up about 20% of the brain volume^36^. Hence, a change of a few mM of potassium can lead to a significant relative change of [K^+^]_e_, while barely affecting [K^+^]_i_. As the membrane potential is determined by the ratio between intra- and extracellular ions, the contribution of potassium to the membrane potential is nearly exclusively governed by [K^+^]_e_^37^. Therefore, most studies investigating K^+^ alterations in response to neuronal activity measured extracellular rather than intracellular changes. For example, strong, tetanic neuronal stimulation or epileptic seizures reliably lead to increases of [K^+^]_e_ that plateau around 10 to 12 mM^38,39^, likely because of efficient potassium clearance via astrocytes^39,40^. Cortical spreading depolarizations can further increase extracellular K^+^ levels to around 65 mM^38,41,42^. In contrast, strong tetanic stimulation of frog motor neurons led to a decrease of intracellular [K^+^] by not more than 5 mM^43^, while Raimondo et al*.* estimated that epileptic seizures lead to a decrease of intracellular potassium concentrations of only 2 mM^44^. The relatively small activity-dependent changes in intracellular potassium are even more relevant when considering spontaneous neuronal activity. A single action potential leads to an increase of extracellular potassium by only 0.2–0.8 mM^45,46^. Hence, changes in [K^+^]_i_ are most likely much smaller.

As the EC_50_ of lc-LysM GEPII 1.0 (27–60 mM) is far from the intracellular K^+^ concentration in neurons (~ 140 mM), the sensor will either be close to saturation or fully saturated when expressed in the intracellular space. Indeed, lc-LysM GEPII 1.0 is saturated at 150 mM potassium and a drop to 100 mM leads to a change of the dynamic range by only about 10%^21^. Therefore, small changes of intracellular K^+^ are unlikely to elicit a change of the FRET ratio of lc-LysM GEPII 1.0 strong enough to be resolved. We conclude that to resolve intracellular K^+^ changes in response to spontaneous neuronal activity, lc-LysM GEPII 1.0 needs to be improved towards lower affinity for K+.

Combining live cell imaging with optogenetic stimulation can be used to control [K^+^] changes tightly and therefore allows comparing the performance of potential candidate variants. This might help facilitating the development of improved potassium sensors focusing on resolving K^+^ changes during neuronal activity. The inability to measure [K^+^]_i_ following single action potentials can be attributed to the anticipated little changes in [K^+^]_i_ in such scenarios and the very low EC_50_ of lc-LysM GEPII 1.0 for neuronal measurements.

Despite these shortcomings of lc-LysM GEPII 1.0 for the measurement [K^+^]_i_ in neurons, we were able to express lc-LysM GEPII 1.0 in cortical neurons at a sufficiently high level to be visualized by in vivo imaging and to perform the first measurements of [K^+^]_i_ in the living mouse brain. Like in cultured cells, we did not detect any changes of [K^+^]_i_ during spontaneous neuronal activity. However, we observed increased fluorescence lifetime (FLIM) of lc-LysM GEPII 1.0 during massive neuronal depolarizations triggered by cerebral ischemia. Other groups already used imaging approaches to measure [K^+^] in response to cortical depolarization waves in vivo, however, only in the extracellular space. Transient extracellular K^+^ increases were recorded after injection of the K^+^-sensitive dye APG-2 in the cisterna magna^47^ or topical application of GINKO2 on the open cortex in mice^29^. Wu and colleagues tried to measure [K^+^]_i_ dynamics in neurons and astrocytes of drosophila in response to induced neuronal activity; however, they reported that their data obtained with the single fluorophore sensor GINKO2, which displays a strong pH dependency, is quite likely confounded by activity-induced acidification artifacts^29^. Also, cortical depolarization waves cause intracellular acidification in neurons^48,49^; however, as discussed above, GEPIIs are considerably more pH stable than GINKO2. Therefore, we do not expect that pH affected our measurements considerably. Future measures with improved GEPIIs in terms of K_D_ and pH stability and a pH-sensor such as pHRed^50^ could help disentangle the actual potassium signal from a potential pH artifact^51^.

Our data show that intracellular K^+^ imaging in neurons is, in principle, possible in vivo. However, the low K_D_ of lc-LysM GEPII 1.0 and the high technical requirements of such measurements (in vivo FLIM) currently limit the practical use of this approach. Hence, optimizing lc-LysM GEPII 1.0 with respect to affinity, dynamic range, and fluorescence intensity is critical to allowing the measurement of intracellular K^+^ to become a standard tool in neuroscience research. We believe such a task is worth pursuing because reliably detecting intracellular K^+^ dynamics in vivo will help investigating neuronal phenomena and other critical neurobiological processes such as astrocytic K^+^ clearance and spatial K^+^ buffering^31,32^.

In conclusion, we provide a first functional characterization of lc-LysM GEPII 1.0 in cortical neurons both in vitro and in vivo. We show that lc-LysM GEPII 1.0 can resolve [K^+^] changes in response to intense neuronal activity, however, fails to detect K^+^ dynamics in response to mild activity or single action potentials. Future optimization of lc-LysM GEPII 1.0, especially with respect to an increased K_D_, is needed for allowing precise K^+^ measurements in physiologic settings.

### Supplementary Information

Supplementary Figures.

## Data Availability

The data generated during the study are available from the corresponding author upon reasonable request. lc-LysM GEPII 1.0 is available from the authors upon request.
