# Supplementary material for: Probing intracellular potassium dynamics in neurons with the genetically encoded sensor lc-LysM GEPII 1.0 in vitro and in vivo
Source: Sci Rep. 2024 Jun 14;14:13753. doi: 10.1038/s41598-024-62993-1 (PMC11178854; doi:10.1038/s41598-024-62993-1)
Supplement: Supplementary file 1 — Supplementary Figures. [file 41598_2024_62993_MOESM1_ESM.pdf]

Probing Intracellular Potassium Dynamics in Neurons with the Genetically Encoded Sensor lc-LysM GEPII 1.0 *in vitro* and *in vivo*

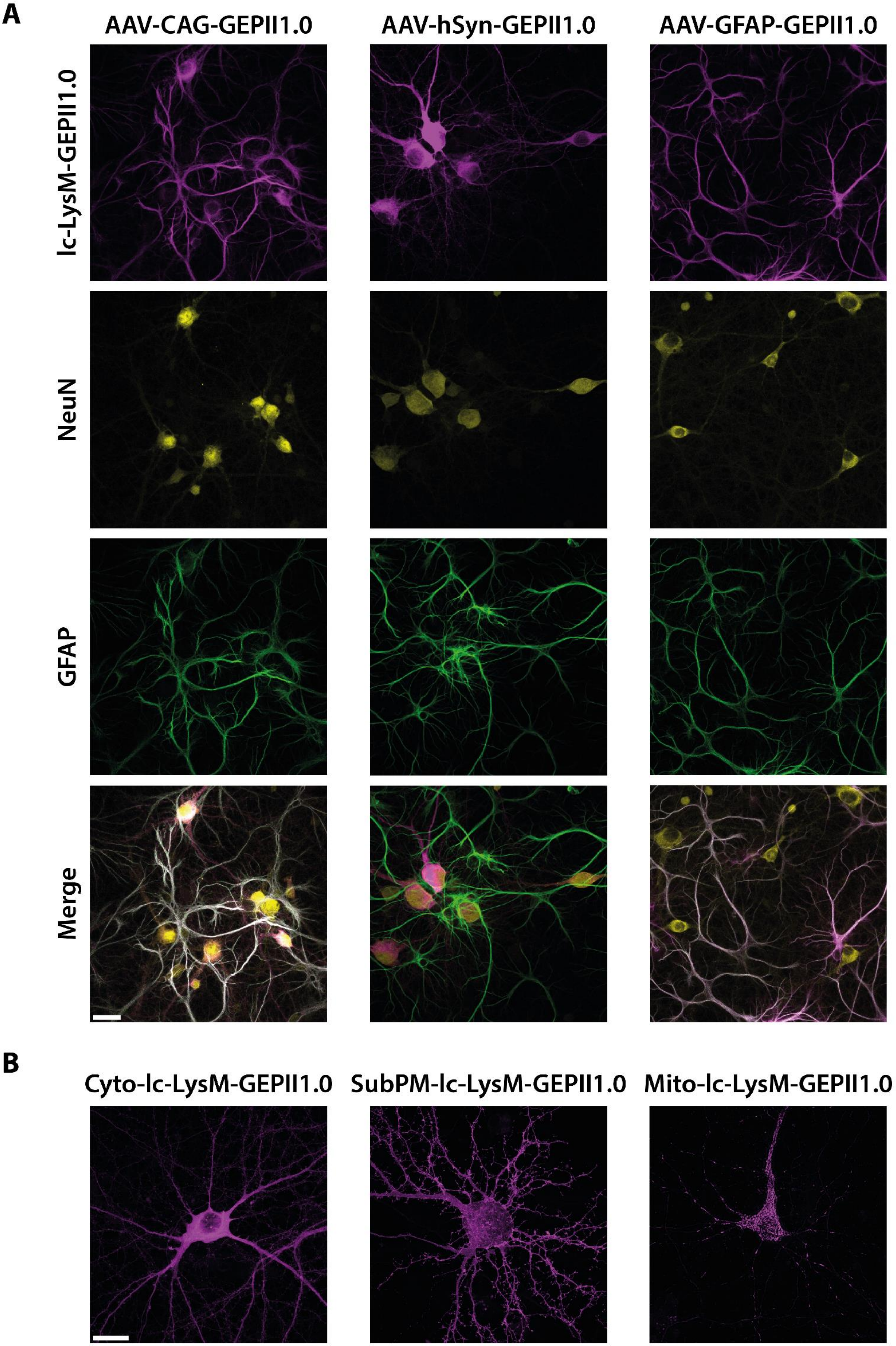
A
AAV-CAG-GEPII1.0
AAV-hSyn-GEPII1.0
AAV-GFAP-GEPII1.0
lc-LysM-GEPII1.0
NeuN
GFAP
Merge
B
Cyto-lc-LysM-GEPII1.0
SubPM-lc-LysM-GEPII1.0
Mito-lc-LysM-GEPII1.0

Supplementary Figure 1: Expression of lc-LysM-GEPII1.0 in different cell types and compartments (A) Mixed cortical cultures containing neurons and astrocytes were transduced with different AAVs to express lc-LysM-GEPII1.0 either ubiquitously (left), specifically in neurons (middle) or in astrocytes (right). The cultures were fixed and counterstained with NeuN and GFAP to confirm cell-type specific expression. Scale bar represents 25 µm. **(B)** Expression of lc-LysM-GEPII1.0 in different subcellular compartments of a neuron. Expression of lc-LysM-GEPII1.0 was targeted either to the cytosol (left), the plasma membrane (middle) or the mitochondrial matrix (right). Scalebar represents 20 µm.

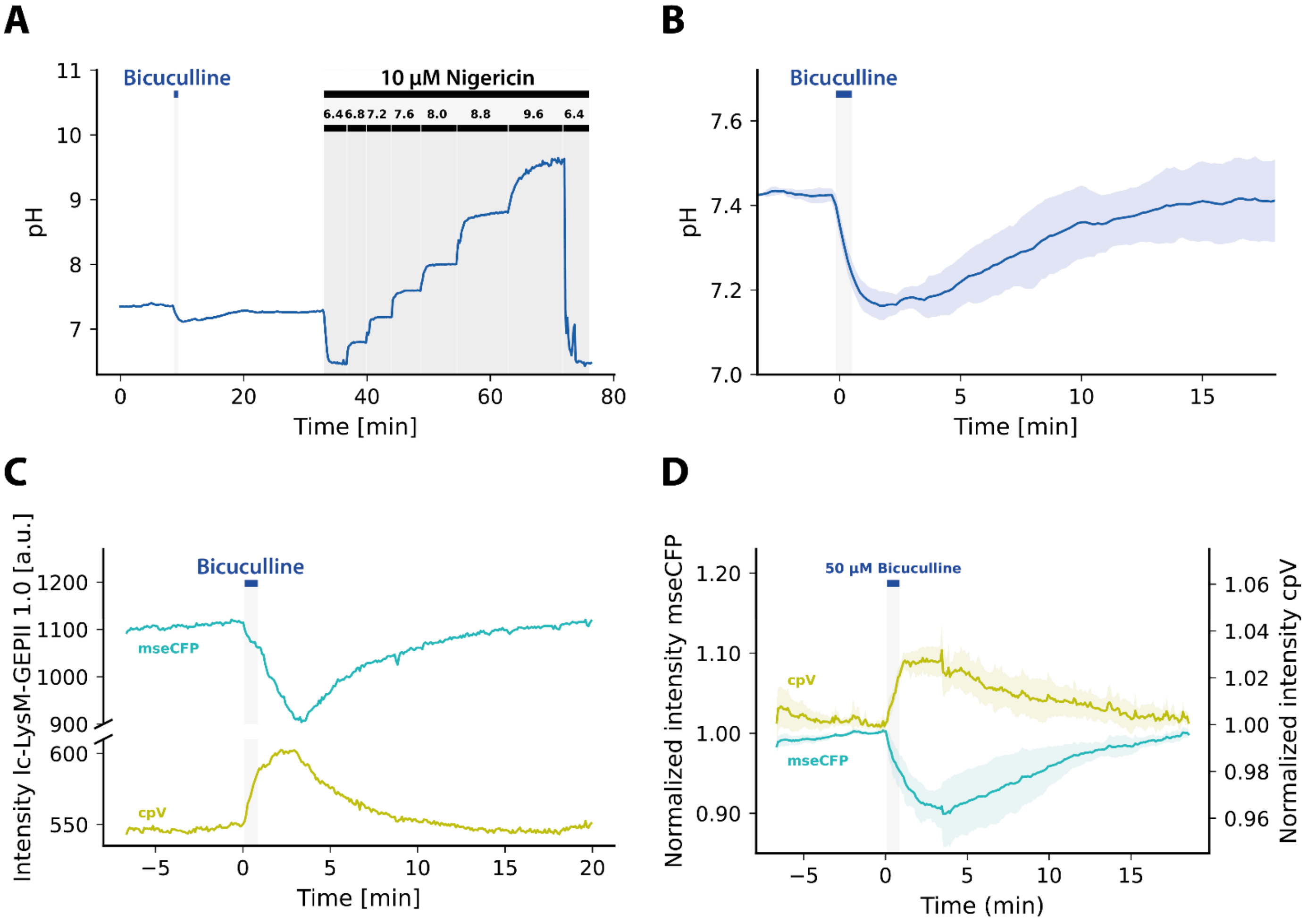

Supplementary Figure 2: Neuronal pH response to Bicuculline (A) Example trace of a neuron expressing SypHer3s in response to 50 µM Bicuculline followed by calibration of SypHer3s in the presence of 10 µM Nigericin. **(B)** Average pH response of neurons to 50 µM Bicuculline. Traces are represented as mean ± SD (N = 3 experiments). **(C)** Example trace of the individual donor and acceptor channels of lc-LysM GEPII 1.0 in response to 50 µM Bicuculline. (D) Average traces of the individual donor and acceptor channels of lc-LysM GEPII 1.0 in response to 50 µM Bicuculline (N = 5 experiments).

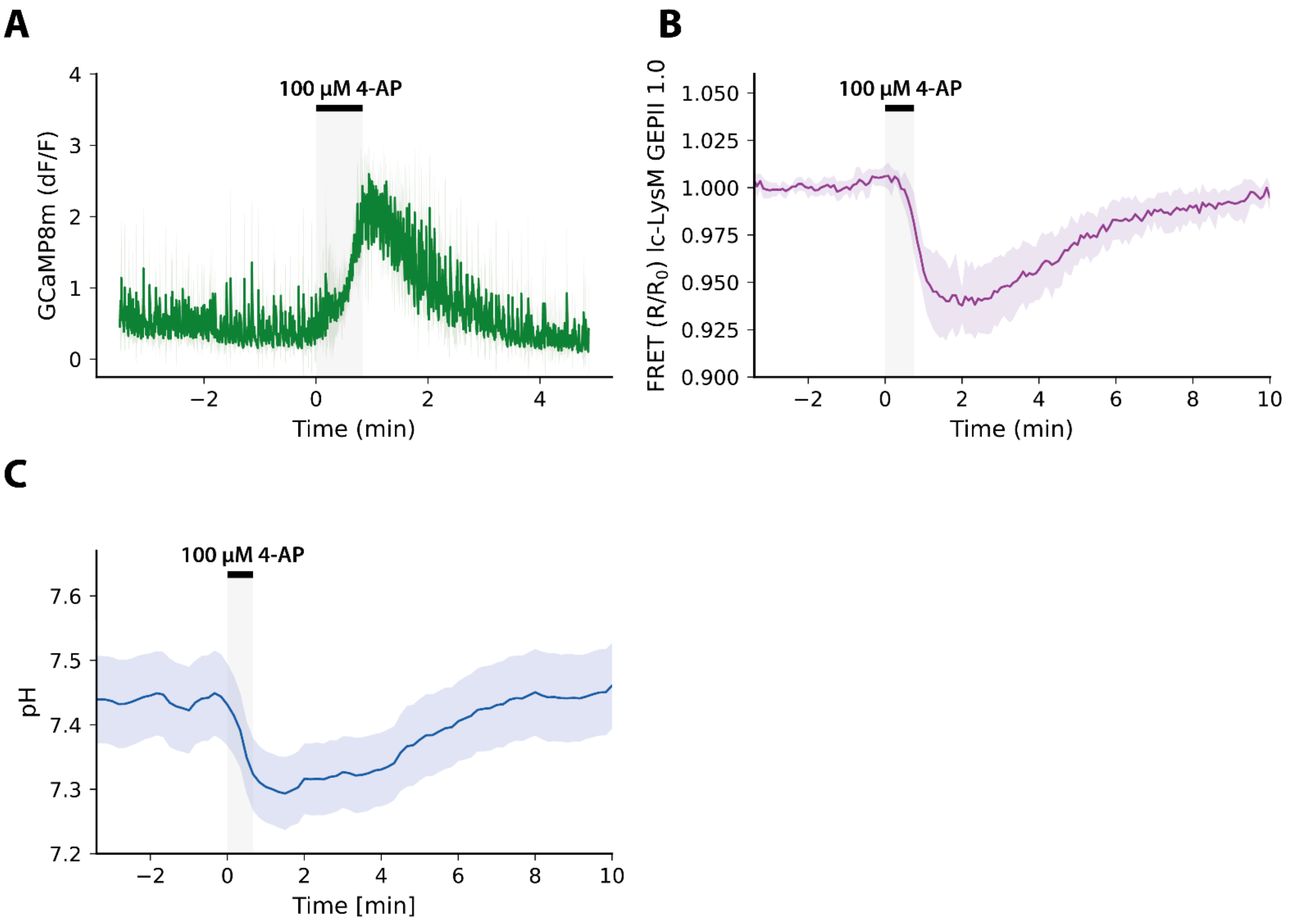

Supplementary Figure 3: Neuronal response to 4-AP (A) Average trace of the mean GCaMP8m response to 100 μM 4-AP (N = 3 experiments). Traces are represented as mean ± SD. **(B)** Averaged trace of the mean lc-LysM-GEPII1.0 response to 100 μM 4-AP (N = 5 experiments). Traces represented as mean ± SD. **(C)** Example trace of the SypHer3s response to 100 μM 4-AP (N = 16 neurons in 1 experiment). Trace represents mean ± SD.

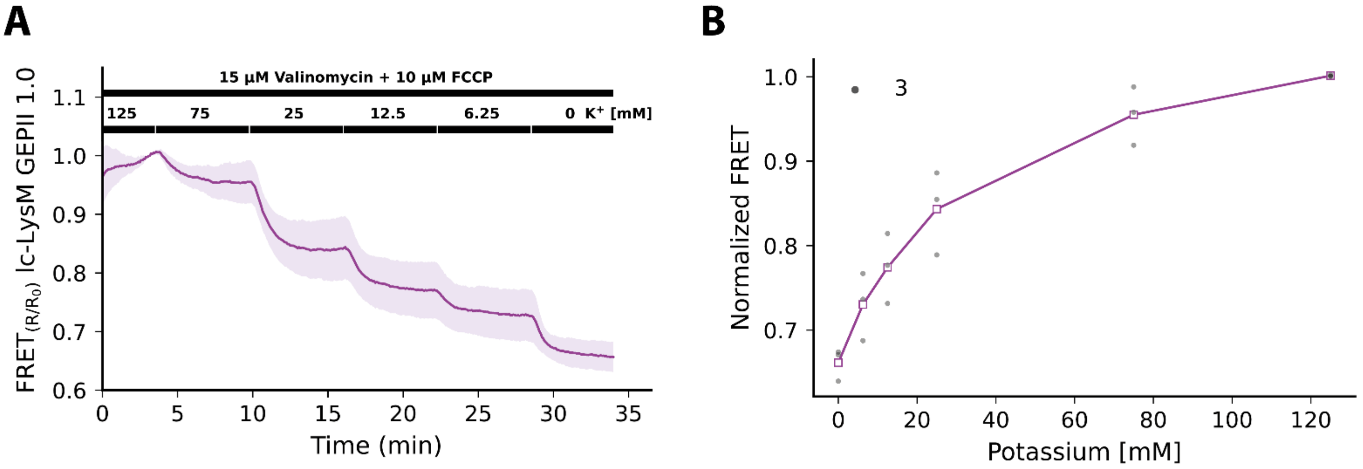

Supplementary Figure 4: Calibration of lc-LysM-GEPII1.0 in permeabilized astrocytes (A) Average trace of lc-LysM GEPII 1.0 during calibration with different concentrations of K^+. Traces are represented as mean ± SD (N = 3 experiments)

(B) Quantification of the FRET ratio of lc-LysM GEPII 1.0 at different K^+ concentrations relative to 125 mM.

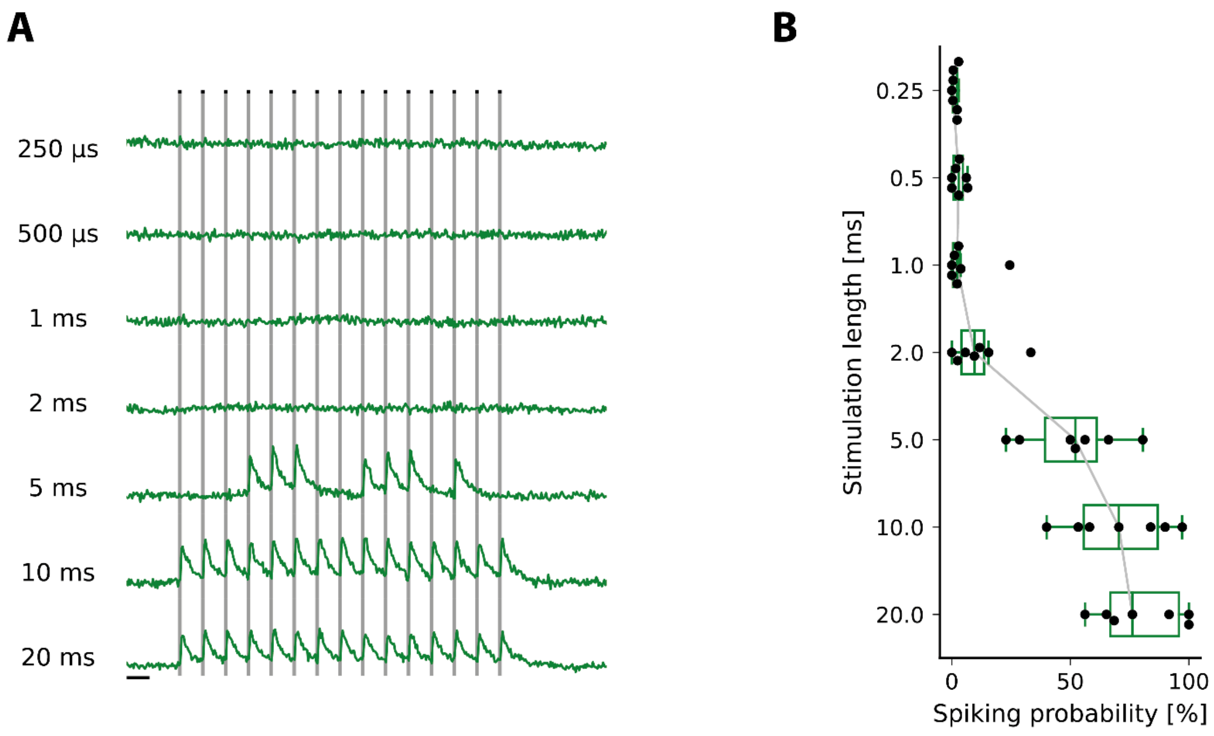

Supplementary Figure 5: Minimal pulse length of optogenetic stimulus for reliable stimulation (A) Example trace of an individual neuron expressing GCaMP8m and ChrimsonR-tdT. Stimulation trains containing 15 stimuli at 0.5 Hz were delivered. Individual stimuli between different stimulation trains increased in duration of the individual pulse length from 250 µs to 20 ms. Scale bar represents 2 seconds. **(B)** Quantification of the spiking probability in response to different pulse lengths. Spiking probability was calculated per cell as number of spikes correlating with the stimulation divided by the amount of stimuli (e.g. 15). Each data point is the mean spiking probability of all cells per experiment. Data is represented as mean ± SD. N = 7 Experiments.

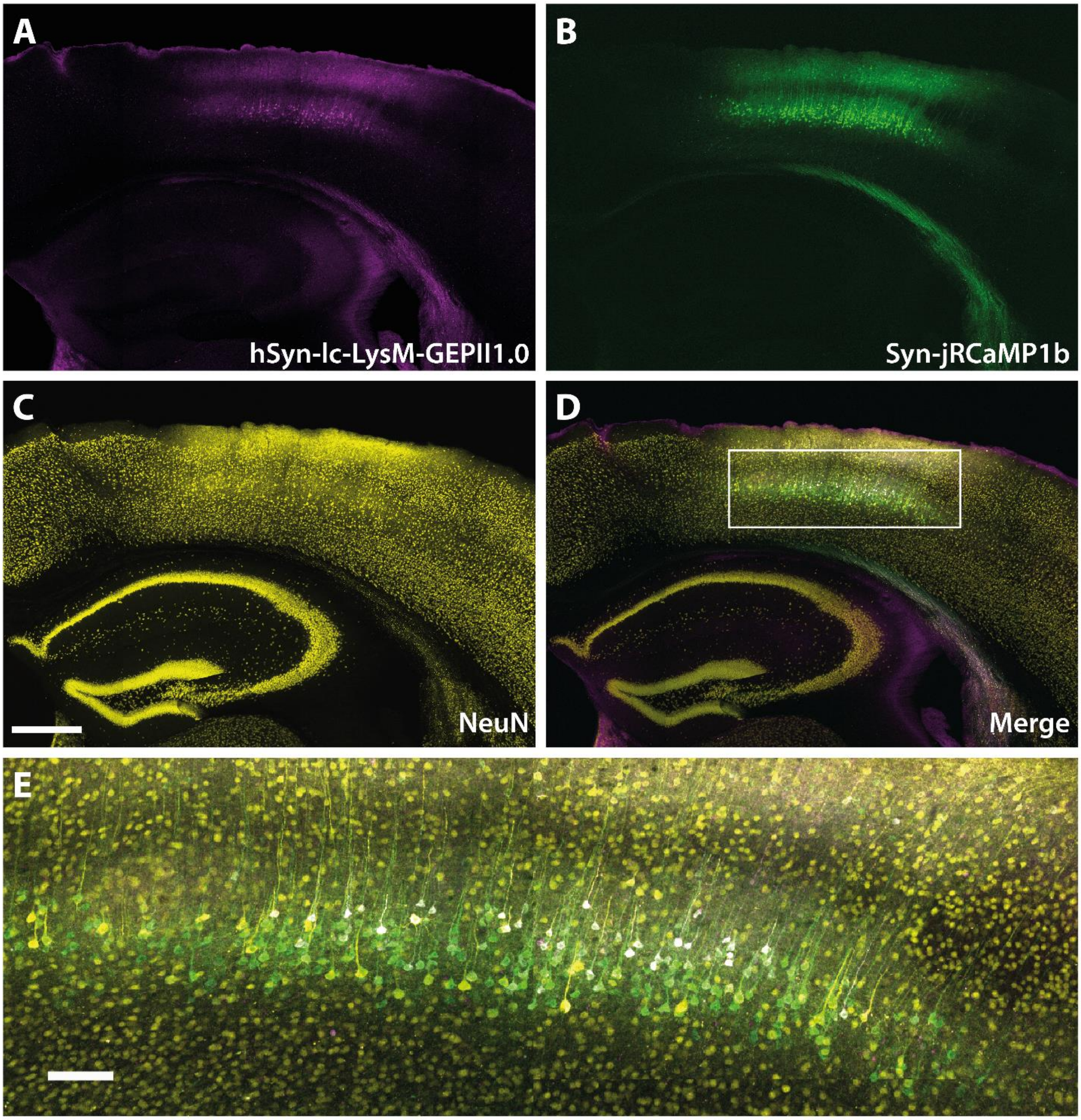

Supplementary Figure 6: Expression of lc-LysM-GEPII1.0 and jRCaMP1b in neurons in the cortex of mice. Maximum intensity projections of brain slice overviews of neurons in layer 5 after viral labelling with lc-LysM-GEPII1.0 **(A)** and jRCaMP1b **(B)**. Neuronal cell bodies were counterstained with NeuN **(C)**. Scale bar represents 500 µm. **(D)** Both sensors overlap with the NeuN signal and confirm expression of the sensors in neurons. **(E)** Magnification of region indicated in (D). Scalebar represents 100 µm.

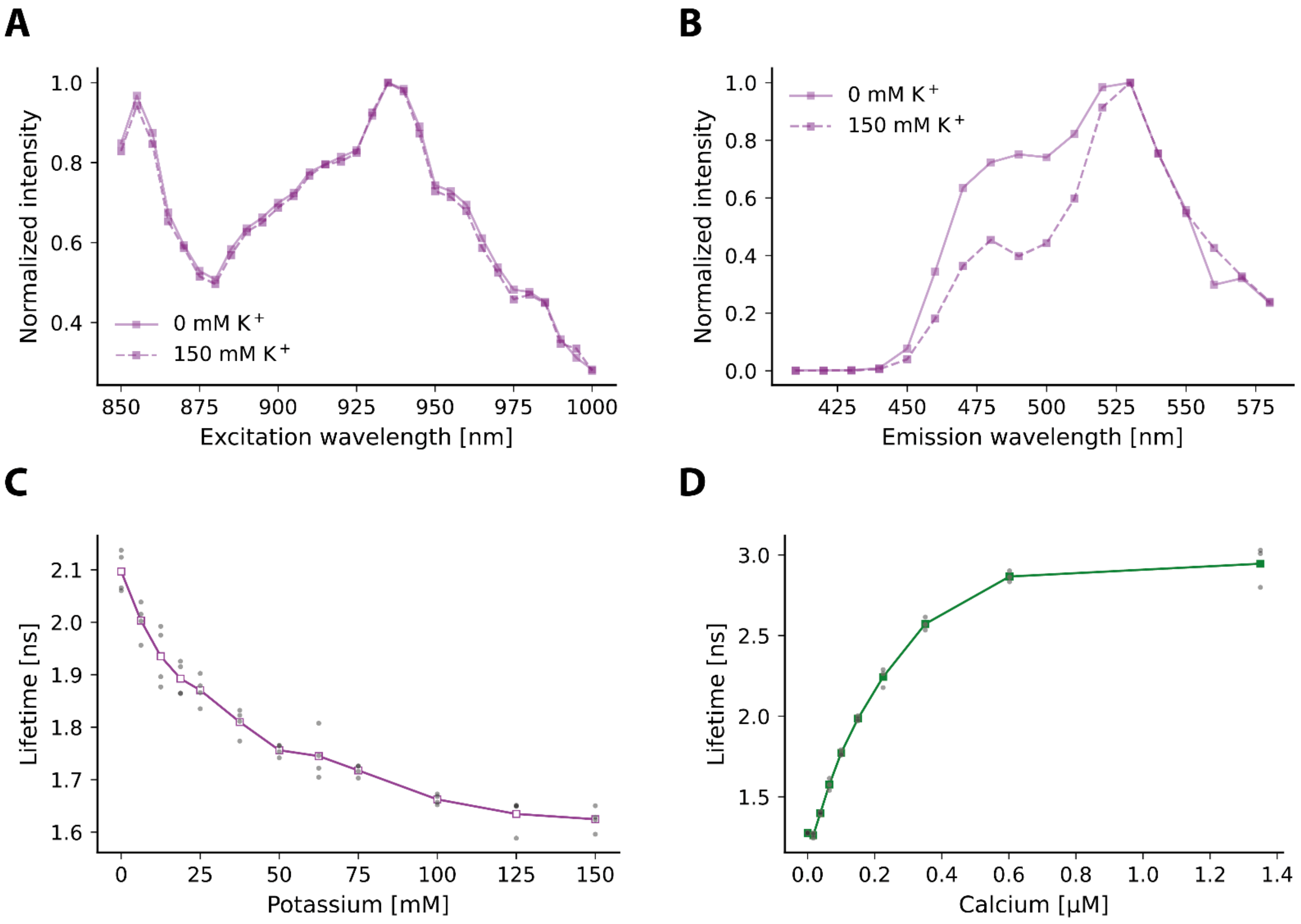

Supplementary Figure 7: 2-Photon Excitation and emission spectra of lc-LysM-GEPII1.0 and FLIM calibrations of lc-LysM-GEPII1.0 and jRCaMP1b All presented data were acquired using recombinant protein of the respective sensor in a 10 mM HEPES solution containing different concentrations of potassium. To keep the osmolarity of the solution constant, it always contained a total of 150 mM of NaCl and KCl. When KCl was reduced, NaCl was increased accordingly. **(A)** 2-Photon excitation spectrum of lc-LysM-GEPII1.0 in the presence of 0 or 150 mM K^+. **(B)** Emission spectrum of lc-LysM-GEPII1.0 in the presence of 0 or 150 mM K^+. Excitation was at 850 nm. **(C)** FLIM calibration of lc-LysM-GEPII1.0 with different concentrations of K^+. Data is represented as mean. n = 4 Experiments **(D)** FLIM calibration of jRCaMP1b with different concentrations of Ca^{2+}. Data is represented as mean. n = 3 Experiments
